# Optical and Photosensitive Properties of Flexible *n* (*p*)–InSe/In_2_O_3_ Heterojunctions

**DOI:** 10.3390/ma15093140

**Published:** 2022-04-26

**Authors:** Veaceslav Sprincean, Liviu Leontie, Iuliana Caraman, Dumitru Untila, Mihaela Girtan, Silviu Gurlui, Petru Lisnic, Corneliu Doroftei, Aurelian Carlescu, Felicia Iacomi, Mihail Caraman

**Affiliations:** 1 Applied Physics and Informatics Department, Faculty of Physics and Engineering, Moldova State University, A. Mateevici, 60, MD-2009 Chisinau, Moldova; sprincean@gmail.com (V.S.); dumitru.untila.andrei@gmail.com (D.U.); mihailcaraman@yahoo.com (M.C.); 2 Faculty of Physics, Alexandru Ioan Cuza University of Iasi, Blvd. Carol I, No. 11, 700506 Iasi, Romania; lisnic.petru@yahoo.com (P.L.); iacomi@uaic.ro (F.I.); 3 University of European Political and Economic Studies “Constantin Stere”, Stefan cel Mare si Sfint Blvd., 200, MD-2004 Chisinau, Moldova; iucaraman@yahoo.ca; 4 Photonics Laboratory, (LPhiA) E.A. 4464, SFR Matrix, Faculty of Sciences, Angers University, 2 Bd Lavoisier, 49045 Angers, France; mihaela.girtan@univ-angers.fr; 5 Research Center in Environmental Sciences for the North-Eastern Romanian Region (CERNESIM), Science Research Department, Institute of Interdisciplinary Research, Alexandru Ioan Cuza University of Iasi, Bulevardul Carol I, No. 11, 700506 Iasi, Romania; corneliu.doroftei@uaic.ro

**Keywords:** chalcogenides, heterojunctions, thin films, single crystals, optical properties, photoluminescence, photosensitivity

## Abstract

In this work, optical, including photoluminescence and photosensitivity, characteristics of micrometer-sized flexible *n* (*p*)–InSe/In_2_O_3_ heterojunctions, obtained by heat treatment of single-crystalline InSe plates doped with (0.5 at.%) Cd (Sn), in a water-vapor- and oxygen-enriched atmosphere, are investigated. The Raman spectrum of In_2_O_3_ layers on an InSe:Sn substrate, in the wavelength range of 105–700 cm^−1^, contains the vibration band characteristic of the cubic (bcc-In_2_O_3_) phase. As revealed by EDX spectra, the In_2_O_3_ layer, ~2 μm thick, formed on InSe:Cd contains an ~18% excess of atomic oxygen. The absorption edge of InSe:Sn (Cd)/In_2_O_3_ structures was studied by ultraviolet reflectance spectroscopy and found to be 3.57 eV and ~3.67 eV for InSe:Cd and InSe:Sn substrates, respectively. By photoluminescence analysis, the influence of doping impurities on the emission bands of In_2_O_3_:Sn (Cd) was revealed and the energies of dopant-induced and oxygen-induced levels created by diffusion into the InSe layer from the InSe/In_2_O_3_ interface were determined. The *n* (*p*)–InSe/In_2_O_3_ structures display a significantly wide spectral range of photosensitivity (1.2–4.0 eV), from ultraviolet to near infrared. The influence of Cd and Sn concentrations on the photosensitivity and recombination of nonequilibrium charge carriers in *n* (*p*)–InSe layers from the heterojunction interface was also studied. The as-obtained nanosized InSe/In_2_O_3_ structures are suitable for optoelectronic applications.

## 1. Introduction

Indium monoselenide (InSe) is a typical representative of group III–VI layered materials (In and Ga monochalcogenides). Its single crystals are comprised of elementary stratified Se–In–In–Se packages, bound by van der Waals forces, much weaker than those between four monoatomic sheets inside a package, corresponding to an ionic-covalent (predominant) bonding [1]. This allows displacement of neighboring packages relative to each other, which explains InSe polytypism and flexibility.

Being an *n*-type material with a room-temperature direct band gap of about 1.3 eV and an indirect one of 1.26 eV, InSe is considered, along with other classes of narrow-gap semiconductors, as a promising material for solar energy applications [2,3]. As a typical feature of InSe and other lamellar III–VI semiconductors (GaS, GaSe, GaTe, etc.), at the interface between stratified chalcogen–metal–metal–chalcogen packages, the valence bonds of chalcogens are practically completely compensated. This leads to a low surface-state density (~10^10^ cm^−2^) and a low recombination rate of nonequilibrium charge carriers, which, together with a high absorption coefficient near the fundamental absorption edge, are requirements for broadband photosensitivity and high energy conversion efficiency of devices based on these materials.

As recent research has shown, by various technological procedures and, especially, by heat treatment in air, at temperatures below the melting point of semiconductor material, thin films of In_2_O_3_ and Ga_2_O_3_ oxides are formed on the surface of InSe and GaSe lamellae, respectively [4,5,6]. Indium (In_2_O_3_) and gallium (Ga_2_O_3_) native oxides are *n*-type semiconductors with a wide band gap, of 3.7 eV and 4.8 eV, respectively, extensively studied due to their wide range of technological applications, especially as electrodes in solar cells and in various optoelectronic devices [7,8].

An important role in the operation of photovoltaic devices is played by their surface reflection capacity. The refractive index of In_2_O_3_ and InSe in the visible–near infrared (vis–NIR) range is 1.9–2.0 and 2.9–3.0, respectively. Thus, the normal-incidence reflection coefficient for the surface of the In_2_O_3_ layer is ~9.6–11%. At the same time, the InSe/In_2_O_3_ pair, for certain thicknesses of the In_2_O_3_ layer, is able to perform an anti-reflective function (reflection factor ≈ 0) over a wide spectral range. These properties have led to various developments in optoelectronics based on lamellar semiconductors and structures with *n*–In_2_O_3_ as an optically transparent electrode [9,10]. The rapid progress in modern optoelectronic technologies has driven the development of new low-dimensional materials with unusual properties (nanowires, nanolamellae) [11], as well as of devices based on them, especially flexible electronic devices, various bio-integrated devices, and many others [12,13,14]. According to recent literature (including the references mentioned above), researchers’ attention is currently focused on the development of ultraviolet optoelectronic devices (UV-C) with a narrow photosensitivity band in the UV region, including the “solar blind” range (solar radiation that is strongly absorbed and cannot reach the earth’s surface) [15,16]. These (solar blind) devices find application in fire alarm/detection systems, missile approach warning systems, biomedical analyses, astrophysics studies, etc. [17].

In this paper, we investigate the chemical composition and optical properties, including photoluminescence (PL) and photosensitivity, of *n*–In_2_O_3_/*p*–InSe:Cd and *n*–In_2_O_3_/*n*–InSe:Cd heterostructures obtained by the heat treatment of *n*–InSe:Sn and *p*–InSe:Cd plates in open air at a temperature of ~600 °C. The ultimate goal of these studies is to develop flexible photoelectric devices with broadband photosensitivity.

## 2. Materials and Methods

To obtain the *n*–In_2_O_3_/*p*–InSe and *n*^+^–In_2_O_3_/*n*–InSe structures, single-crystalline plates of InSe doped with 0.5 at.% Cd (to get *p*–InSe) and 0.5 at.% Sn (*n*–InSe), respectively, were used. Cadmium- and Sn-doped InSe single crystals were grown by the Bridgman–Stockbarger technique. The primary InSe:Cd and InSe:Sn compounds were synthesized from component elements, In (5N) and Se (5N), which were taken in stoichiometric quantities. The respective amounts of In and Se, along with chemical elements Cd (5N) and Sn (5N), in proportions of 0.5 at.%, were introduced into quartz ampoules.

Electron and hole concentrations in *n*–InSe and *p*–InSe single crystals, at room temperature, determined from Hall effect measurements, were found to be of (4–6) × 10^16^ cm^−3^ and 8 × 10^13^ cm^−3^, respectively. The preparation technology of *n* (*p*)–InSe lamellae and *n* (*p*)–InSe/In_2_O_3_ structures is described in [5].

Micro-Raman spectra were recorded using a WITec RA300 microscope (excitation wavelength of 532 nm). The reflection spectra in the UV region were recorded with a Specord M-40 spectrophotometer with a spectral energy resolution of 0.5 meV, equipped with accessories for reflectance measurements at an incidence angle ≤5°. The wavelength-modulated reflection spectra were recorded with spectrophotometric equipment based on an MDR-2 type monochromator with 1200/600 mm^−1^ diffraction grating, in which one of the flat mirrors was replaced with a vibrating mirror made of Al, deposited on a 120-μm-thick Si plate with a surface area of ~30 cm^2^. The vibration frequency of the mirror was 22 Hz. The wavelength resolution provided by monochromator was Δ*λ* = 2.5 Å.

Photoluminescence and photosensitivity spectra were registered by means of photometric equipment, including an MDR-2 monochromator, equipped with a photomultiplier with a multi-alkali [(Na2K)Sb + Cs] photocathode with a quartz window. A quartz tungsten halogen lamp with a power of 150 W was used as the excitation source of the photocurrent in the examined structures (InSe:Cd and InSe:Sn)/In_2_O_3_. The temperature of the W filament was ~3300 K.

The photosensitivity of (InSe:Cd, InSe:Sn)/native oxide structures was excited with monochromatic radiation provided by a DRS-500 filtered mercury lamp (wavelength *λ* = 546 nm), while PL excitation was performed using a N_2_ laser (*λ* = 334 nm) and an MRL-637 red solid-state laser with a 100 mW output power. The intensity of the laser exciting radiation was attenuated using neutral density filters with thin Pt films deposited onto amorphous quartz plates (molten SiO_2_).

The photoresponse of *p*–InSe:Cd/*n*–In_2_O_3_ heterojunctions upon bending at an angle of 15°, in the plane perpendicular to the sample surface, was recorded on a structure formed on a plate of *p*–InSe doped with 0.5 at.% Cd, with a thickness of 28 μm. At the same time, a photoresistor was made based on an InSe:Cd plate with a thickness of 24 μm of the same material. Thin In films were used as electrodes. The distance between them was 6 mm, while the surface area was equal to ~30 mm^2^. The thickness of the plates was calculated from the interference fringes of the transmission spectrum at wavelengths of 5–15 μm. The dependence of the photocurrent on the sample illumination using a (637 nm and 2 mW) laser radiation was studied. The beam density was ~1.5 × 10^16^ photons·cm^−2^.

## 3. Results and Discussion

By heat treatment in air, for 6–12 h, at a temperature of 600 °C, of InSe lamellae, specially undoped and doped with Sn and Cd, their surface is covered with a purple-blue In_2_O_3_ layer, displaying a poor diffuse reflection. The surface morphology of these layers was studied in [18]. The performed scanning electron microscopy (SEM) inspection revealed that the In_2_O_3_ layer is composed of nanowires and nanoribbons, the length of which is tens of nanometers [5].

The X-ray diffraction (XRD) patterns of the materials obtained by heat treatment, in air, of plates with a thickness of 10–30 μm contain intense diffraction lines of the native oxide (In_2_O_3_), together with lines of a much lower intensity of the primary material (InSe). Valuable information on the structure and chemical composition of layers formed on semiconductor and dielectric surfaces can be obtained from X-ray photoelectron spectroscopy (XPS) and energy dispersive X-ray spectroscopy (EDX) analyses [19,20]. These surface analysis techniques are based on the fact that the binding energy of atom inner shell electrons varies depending on the chemical state of the neighboring atoms. The higher the electronegativity of the neighboring atoms, the higher the ionization energy of the inner shells. Although the energy resolution of EDX spectroscopy is lower than that of XPS and auger electron spectroscopy (AES), the EDX analysis can still be used to determine the elemental composition of thin metal (Ga, Zn, In) oxide layers [18,21,22,23,24].

**Figure 1:**
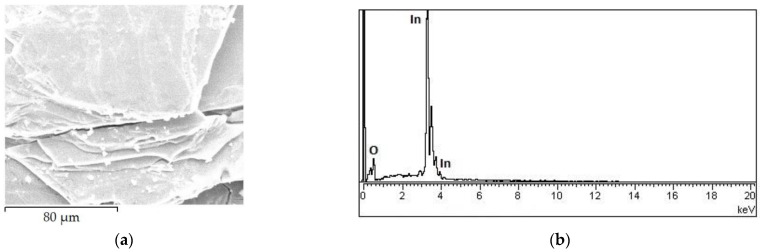
SEM image (**a**) and the EDX spectrum (**b**) of the In_2_O_3_ layer formed on the surface of the InSe:Cd (0.5 at.%) plate.

Figure 1a shows the SEM micrograph for the region in close vicinity of the edge of the InSe:Cd plate (thickness 180 μm), heat-treated in air at 600 °C for 6 h. As can be clearly seen from this image, cracks and micro-defects are present on the surface oxide layer of InSe:Cd plates. The elemental composition of the layer formed on the plate surface was determined by the intensity of the characteristic lines in the EDX spectrum (Figure 1b).

As can be seen from this figure, the layer of material penetrated by the electron beam with an energy of 20 keV contains indium and oxygen. SiO_2_ and InAs were used as standards for determining the concentration of O and In atoms. Their concentrations (Figure 1b) were found to be 22.45 wt.% and 67.51 at.% for O (K) and 77.55 wt.% and 32.49 at.% for In (L). If it is admitted that all the In atoms form the compound In_2_O_3_, then the layer of analyzed material contains a surplus of ~16% atomic oxygen.

In [24], nanostructured In_2_O_3_ (in the form of nanowires and nanoprisms) was obtained by the calcination of In(OH)_3_ micro-crystallites at 350 °C for 4 h. The In/O concentration ratio was found to be 2/3, which corresponds to the stoichiometric composition In_2_O_3_. One can admit that the surplus of oxygen atoms in the In_2_O_3_ layer on the InSe substrate is determined by the high oxygen absorption capacity (from air) of the In_2_O_3_ nanowires/nanopangles formed at the temperature of 600 °C.

The depth (*d*) of the In_2_O_3_ layer penetrated by the electron beam with energy *E*_0_ = 20 keV can be approximated using the Kanaya–Okayama empirical formula [25]:

(1) $$d\;(\mu m)=\frac{0.0276}{\rho }\frac{A}{Z^{0.89}}E_{0}{}^{1.67},$$

where *Z* is the atomic number, *A* denotes the mass number, and *ρ* is the density (g/cm^3^). For *ρ* = 7.2 g/cm^3^, *Z* = 122, *A* = 278, and *E*_0_ = 20 keV, *d* = 2.2 μm.

**Figure 2:**
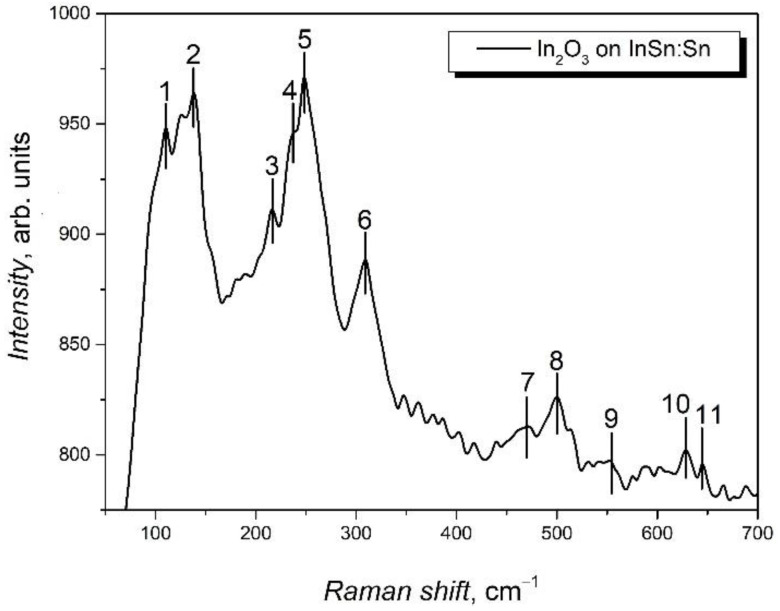
Raman spectrum of the In_2_O_3_ layer obtained by 6 h heat treatment in air, at 600 °C, of single-crystalline InSe plates doped with Sn (InSe:Sn/In_2_O_3_ structures).

Additional information concerning the chemical composition of the layer formed on the surface of the InSe:Sn plates was obtained from the analysis of Raman spectra (Figure 2). In the wavenumber range of 60–700 cm^−1^, eight vibration bands are well emphasized, of which four intense bands are located at low frequencies, 100–300 cm^−1^. In refs. [26,27,28], the peak at 135 cm^−1^ is associated with the In–O vibrations in the InO_6_ octahedron, while the peak located at 306 cm^−1^ is attributed to the stretching vibrations of the octahedra. The 501 cm^−1^ and 627 cm^−1^ high-frequency vibration modes are interpreted in [29] as stretching vibrations in the InO_6_ octahedra. As can be seen from Figure 2, in the spectral range of 340–450 cm^−1^, some vibration bands are missing and, instead, an intense peak at 250 cm^−1^ is present. In the micro- and nanostructured In_2_O_3_ layers with oxygen vacancies, a low-intensity Raman peak centered at 306 cm^−1^ is clearly emphasized [30]. The presence of the 250 cm^−1^ peak for the In_2_O_3_ layers on the InSe substrate may be caused by the excess oxygen in it.

In order to further study the chemical composition of the layer formed on the surface of the InSe:Sn plate, the Raman peak positions (in cm^−1^) from Figure 2 are summarized in Table 1. For comparison, the vibration frequencies of the rhombohedral InSe lattice [31], as well as those of the body-centered cubic In_2_O_3_ in the form of nanowires [32] and nanocubes [30], were also included. As can be seen from this table, the intense bands with the maxima at 110, 135, 231, 306, and 627 cm^−1^ (Figure 2) are in good agreement with the Raman frequencies in the ensembles of In_2_O_3_ nanoformations, identified in the works [30,32] as vibrations in cubic In_2_O_3_ crystallites. The good correlation between the sets of vibration modes of the In_2_O_3_ layers (Table 1, column 2) and those of the bulk In_2_O_3_ single crystals (column 6) indicates that both In_2_O_3_ nanowires and crystallites are present in the layer formed on the surface of the InSe:Sn plate. At the same time, the low-intensity bands positioned at 225, 475, 540, and 635 cm^−1^ (Table 1, column 7) are emphasized in the Raman spectrum, which have been identified in [33] as surface vibration modes in nanocrystalline SnO_2_. The 1–2 cm^−1^ difference between the Raman peak frequencies in the In_2_O_3_ layers formed on the surface of the InSe:Sn plates and those in nanowires and nanocubes can be caused by the nature and size of the In_2_O_3_ nanoformations.

**Figure 3:**
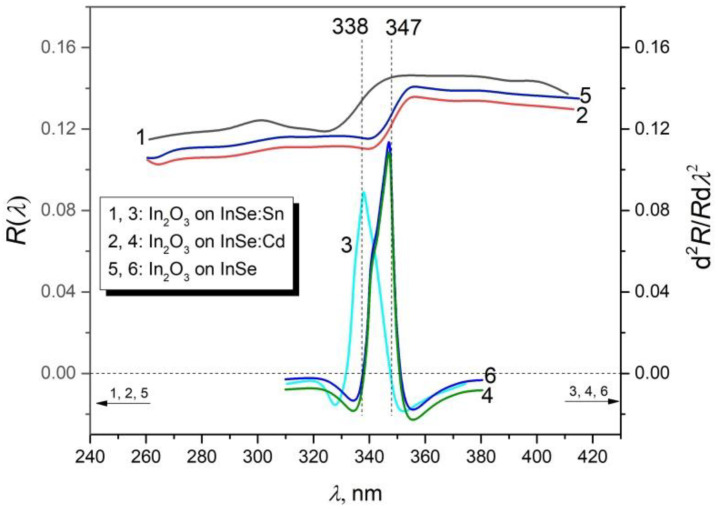
Reflection spectra of the In_2_O_3_ layer on InSe:Sn (curve 1), InSe:Cd (curve 2), and undoped InSe (curve 5) substrates and their second derivatives with respect to wavelength (curves 3, 4, and 6, respectively).

Figure 3 shows the reflection spectra for an incidence angle of ~5°, in the region of the fundamental absorption edge of In_2_O_3_ layers on *n–* and *p*–InSe substrates.

The reflection factor, *R*, at normal incidence, for the separation surface between two distinct optical media depends on the relative refractive and extinction indices, *n_r_* and *k_r_*, respectively, by means of the relation [34]:

(2) $$R=\frac{{\left(n_{r}-1\right)}^{2}+k_{r}{}^{2}}{{\left(n_{r}+1\right)}^{2}+k_{r}{}^{2}}.$$

In the optical transparency region (*λ* > 380 nm), the inequality *k_r_*^2^ << (*n_r_* – 1)^2^ is valid so that the term *k_r_*^2^ can be neglected in both the numerator and the denominator of the above equation [35,36]. The refractive index of the In_2_O_3_ layer depends on the electron concentration and varies between 2.17 and 1.83 for concentrations in the range of 10^19^ < *N_e_* < 10^21^ cm^−3^ [37]. In the considered case, putting *n_r_* ≈ 2 in Equation (2), a reflection factor of ≈11% is obtained for the air/In_2_O_3_ interface.

As can be seen from Figure 3 (curves 1 and 2), in the vicinity of the fundamental absorption edge of In_2_O_3_ crystallites, an increase by 2–3% in the reflectance can be observed together with an increasing wavelength. As can be seen from Figure 3 (curves 3 and 5), in the wavelength range of 300–360 nm, the reflection spectrum of the In_2_O_3_ layer on the undoped InSe substrate overlaps with that recorded for the In_2_O_3_ layer formed on the doped substrate with 0.5 at.% Cd.

The linear region of the reflectance graphs for the In_2_O_3_ layer formed on InSe:Cd plates exhibits an ~15 nm redshift with respect to that formed on the surface of InSe:Sn plates. This displacement is clearly emphasized in the second derivative of the reflectance spectra, d^2^*R*(*λ*)/*R*d*λ*^2^.

The energy band gap in semiconductor materials can be suitably determined from modulated reflection spectra (by wavelength, electric field, temperature, etc.), considering the wavelength at which the function (∆*R/R*) (*λ*) passes through zero [38]. In Refs. [36,39], the direct and indirect band gaps are determined from the spectral analysis of the function (∆*R*/*R*∆*λ*) (*λ*) in van der Waals and III–VI (GaS, GaSe, InSe) crystals. Since the photon energy for which this function passes through zero cannot be accurately determined, the property of the second derivative d^2^*R*(*λ*)/*R*d*λ*^2^ to reach its maximum (a well-pronounced peak) at a photon wavelength corresponding to the forbidden bandwidth was used. The analysis method for the particularities of the reflection spectra by means of the d^2^*R*(*λ*)/*R*d*λ*^2^ function elaborated in [35] is widely applied in the analysis of the FTIR reflection spectra.

In Figure 3 (curves 3, 4, and 6), plots of the second derivative, d^2^*R*(λ)/*R*d*λ*^2^, are also presented. The maxima of respective functions are positioned at 338 nm (3.69 eV) and 347.2 nm (3.57 eV) for the reflection spectra from the In_2_O_3_ layer on InSe:Sn, InSe:Cd, and undoped InSe substrates, respectively. In [40], it was established that the band gap of In_2_O_3_ layers increases from 3.55 to 3.80 eV with increasing concentration of free charge carriers, from 2.6 × 10^19^ to 7.0 × 10^19^ cm^−3^. At the same time, the electron effective mass is also increasing (>0.8 *m*_0_) with the electron concentration in In_2_O_3_ for *n* ≥ 10^19^ cm^−3^ [41].

Indium oxide is an *n*–type semiconductor. Its doping with Sn contributes to the increase in the concentration of free charge carriers, while Cd as a dopant compensates the free charge carriers, thus decreasing the electron concentration in the conduction band (CB). One can consider that at low concentrations, Cd doping does not practically influence the optical band gap, *E_g_*_0_ (Figure 3, curve 6). The direct band gap in a heavily doped semiconductor, *E_gn_*, is given, in virtue of the Burstein–Moss model (parabolic band approximation), by [42]:

(3) $$E_{gn}=E_{g0}+\frac{\hbar^{2}}{2m_{\mathrm{vc}}{}^{*}}{\left(3\pi^{2}n\right)}^{2/3},$$

where *E_g_*_0_ is the band gap of the undoped material; *m*_vc_^∗^ denotes the reduced effective mass of charge carriers (1/*m*_vc_^∗^ = 1/*m*_v_^∗^ + 1/*m*_c_^∗^) [43], with *m*_v_^∗^ and *m*_c_^∗^ representing valence band (VB) and CB effective mass, respectively; *ħ* is the reduced Planck’s constant; and *n* is the electron concentration. Since *m*_vc_^∗^ ≥ *m*_0_ [44], one can consider *m*_vc_^∗^ = *m*_c_^∗^. If one admits that the optical band gap of In_2_O_3_ corresponds to that of the In_2_O_3_:Cd layer (3.57 eV), then from Equation (3), with *m*_vc_^∗^ = 0.2 *m*_0_ (*m*_0_—electron mass) and *E_gn_* = 3.69 eV, the electron concentration in the surface native oxide layer of InSe:Sn plates can be determined and is found to be 2.8 × 10^19^ cm^−3^. The electron concentration in the thin In_2_O_3_:Sn layer, determined by the Hall effect and electrical conductivity measurements, varies between 1.3 × 10^19^ and 1.5 × 10^21^ cm^−3^ [37].

**Figure 4:**
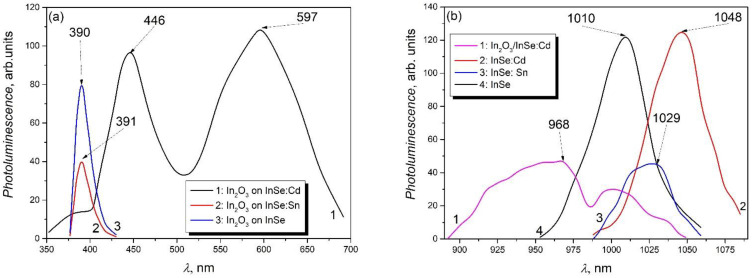
(**a**) Photoluminescence of In_2_O_3_ layers on InSe:Cd (curve 1), InSe:Sn (curve 2), and undoped InSe (curve 3) substrates. (**b**) PL spectra of the material formed on the InSe plate, from the InSe:Cd/In_2_O_3_ interface (curve 1), of InSe:Cd (curve 2), InSe:Sn (curve 3), and undoped InSe (curve 4) single crystals.

The In_2_O_3_ layers formed on *n*–InSe:Sn and *p*–InSe:Cd substrates are materials displaying visible photoluminescence (Figure 4a). Under laser excitation (power density 20 mW/cm^2^) corresponding to the fundamental absorption edge of the In_2_O_3_ nanocrystallite layer on a *p*–InSe:Cd substrate (*λ* = 337.4 nm (3.68 eV)), the PL spectrum (Figure 4a, curve 1) is composed of two intense bands with maxima at 440 nm (2.75 eV) and 590 nm (2.10 eV), and a low-intensity plateau located in the wavelength range of 370–400 nm, with the edge at ~380 nm (3.22 eV).

Photoluminescence of micro- and nanoformations in the visible region has been the subject of many papers, especially [21,24]. The PL spectra of In_2_O_3_ microcrystals cover the wavelength range of 340–500 nm, with maxima positioned at 436 nm and 447 nm, as well as a plateau at 386 nm [24]. At the same time, the PL spectrum of an ensemble of nanospheres, studied in [21], under 400 nm wavelength excitation, is composed of three bands, with peak intensities at 452, 473, and 544 nm, while at 370 nm excitation, the peak intensity of the first two bands changes, while the third band shifts to longer wavelengths by 33 nm. The structure of the PL spectrum and the energy position of the PL maxima depend on the excitation wavelength [21]. The complexity of these PL spectra in the purple–blue region is explained by different concentrations of oxygen vacancies in studied samples [21,24]. The orange–yellow PL band, with a maximum at 580–590 nm, has been observed in many papers in which different types of In_2_O_3_ nanoformations have been studied [18,45,46] and is attributed to radiative transitions from the deep oxygen-vacancy defect energy levels. It is known that In_2_O_3_, in various types of nanoformations, is a good gas (especially oxygen) absorber [47].

The PL spectrum of the In_2_O_3_ layer obtained by heat treatment in air, at 550 °C, for 6 h, of the undoped InSe plates (Figure 4a, curve 3) is analogous to the PL spectrum (curve 2) of the In_2_O_3_ layer on an InSe:Sn substrate; it consists of a weakly asymmetric band with the maximum at 391 nm (3.17 eV). This PL band also predominates in the spectra of In_2_O_3_ nanoparticle ensembles with sizes from units to tens of nanometers [30,48,49].

A narrow band with the maximum located at 388 nm is characteristic of the PL emission of the In_2_O_3_ nanowire layer [48]. A PL spectrum composed of a band located in the wavelength range of 350–440 nm was also obtained for In_2_O_3_ nanocubes. From the comparison of PL spectra of In_2_O_3_ layers obtained by heat treatment in air, at 550 °C, for 6 h, of undoped InSe and 0.5 at.% Cd (Sn)-doped InSe plates (Figure 4), it is clear that the structure of the PL spectra is determined not only by the size and shape of nanoformations and by oxygen vacancies but also by the Cd and Sn impurities, which contribute to the luminescent recombination mechanism in In_2_O_3_.

Figure 4b shows the PL spectra of undoped InSe single crystals (curve 4), InSe doped with 0.5 at.% Cd (curve 2) and Sn (curve 3), as well as the PL spectrum from the InSe:Cd/In_2_O_3_ interface (curve 1), under excitation with 532 nm (2.33 eV) laser radiation with a power density of 5 mW/cm^2^. The PL spectrum of InSe crystals (curve 4) consists of a band with a weak asymmetric contour and the maximum at 1010 nm (1.28 eV), which correlates well with the optical band gap of InSe at room temperature [50]. Therefore, the PL of undoped InSe crystals is determined by the recombination of nonequilibrium charge carriers in the CB with the holes in the VB. The PL peak intensities of InSe single crystals doped with Sn and Cd are located at 1025 nm (1.210 eV) and 1047 nm (1.184 eV), respectively. These maxima exhibit a redshift, compared to the emission band of the undoped crystals, of ~18 meV and 44 meV, respectively. This displacement is likely due to the shift of CB and VB edges in these crystals. The PL band, at 77 K, of Sn-doped InSe single crystals is shifted toward lower energies by 18 meV, compared to that of undoped crystals [51].

The PL spectrum of the material of the interface layer from the InSe:Cd/In_2_O_3_ heterojunction (Figure 4b, curve 1) contains two bands with maxima located at 962 nm (1.287 eV) and 1003 nm (1.235 eV). These bands are shifted toward a high-energy region relative to the PL band of the primary compound (InSe:Cd) (Figure 4b, curve 2) with ~100 meV and 51 meV, respectively. Such a shift can be determined by the presence of different types of nanoformations in the InSe layer from the heterojunction interface. In [52], PL spectra of InSe thin films are provided, from which a blueshift of ~140 meV for film thicknesses smaller than 24 nm can be easily observed.

**Figure 5:**
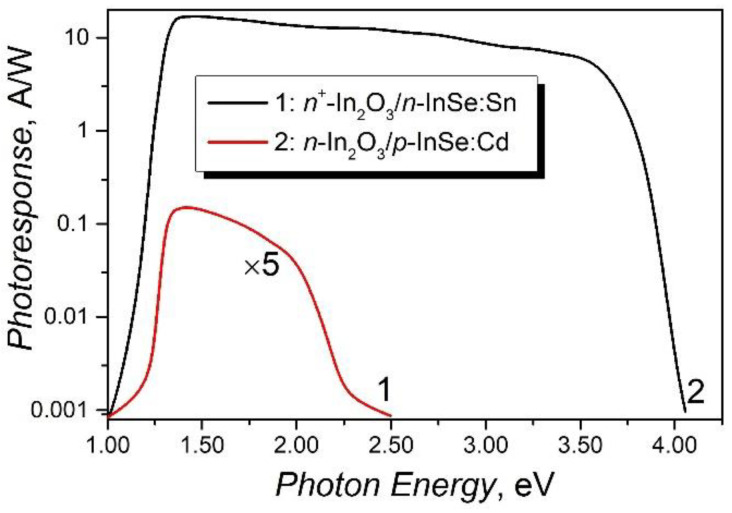
Photoresponse of *n*^+^–In_2_O_3_/*n*–InSe:Sn (curve 1) and *n*–In_2_O_3_/*p*–InSe:Cd (curve 2) heterojunctions as a function of incident photon energy.

Figure 5 shows the spectral dependencies of photoresponse (PR) (photocurrent generated per unit of incident power and unit area of detector) as a function of photon energy for the isotypic structures *n*–InSe:Sn/*n*^+^–In_2_O_3_ (curve 1) and *p*–InSe:Cd/*n*–In_2_O_3_ (curve 2).

The red PR threshold is determined by direct optical transitions in the InSe layer from the interface with In_2_O_3_. Since the In_2_O_3_ layer was formed by the oxidation of the *p* (*n*)–InSe plates, the slow increase in PR together with incident photon energy indicates that at the InSe:Cd/In_2_O_3_ interface, no additional recombination centers of nonequilibrium charge carriers are formed. The mentioned structural features of InSe (and other group III–VI layered materials), together with high absorption coefficient *α* ≥ 1/*L* (*L* is the mean free path of charge carriers), determine the monotonous increase in the photoresponse in heterojunctions with its native oxide (*p*–InSe/In_2_O_3_) and structures based on gallium and indium monochalcogenides [53,54,55]. The abrupt drop in PR at energies higher than 3.65 eV (curve 2) is probably due to the absorption threshold of the In_2_O_3_ layer. Characteristic for heterojunctions formed by semiconductors of the same type is the narrow photoresponse band and the low open circuit voltage [56,57]. The decrease in PR of the *n*–InSe/*n*^+^–In_2_O_3_ structures at energies over 1.90 eV can be determined by the increase in the absorption coefficient of the *n*–InSe:Sn layer and by the presence of an additional concentration of defects at the *n*–InSe/*n*^+^–In_2_O_3_ interface.

The flexibility of the *n*–In_2_O_3_/*p*–InSe:Cd heterojunction and of the *p*–InSe:Cd photoresistor was studied by the repeated (cyclic) bending of the sample by 15°. The radius of curvature was determined by computational fitting of the image of the bent samples and was found to be equal to ~15 cm and 12 cm for the *n*–In_2_O_3_/*p*–InSe:Cd heterojunction and the *p*–InSe:Cd photoresistor, respectively.

**Figure 6:**
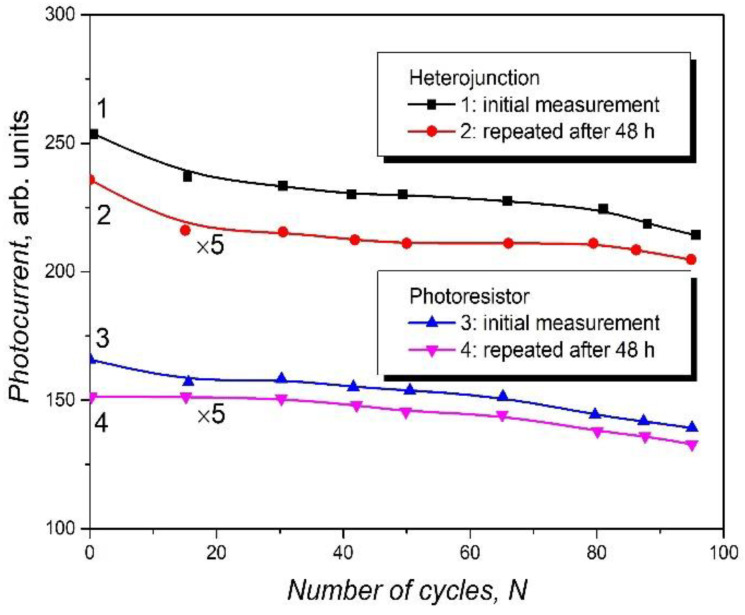
Dependence of the photocurrent as a function of the number of bending cycles (*N*) at 15° for *p*–InSe:Cd/In_2_O_3_ heterojunctions (1, 2) and photoresistor based on the single-crystalline *p*–InSe:Cd plate (3, 4); curves 1 and 3 are the initial measurements; curves 2 and 4 are measurements repeated after 48 h.

Figure 6 shows the photoresponse in the *p*–InSe:Cd/*n*–In_2_O_3_ heterojunction and in the *p*–InSe:Cd lamellar photoresistor, depending on the number of bends in the newly manufactured devices (curves 1 and 3) and for repeated measurements after 48 h (curves 2 and 4). The decrease in the photocurrent at the first bending by 15° can be caused by the decrease in the number of incident photons. Although the monotonous decrease in the photocurrent, of ≤10%, together with the increasing number of deformations from 2 to 90, is probably determined by the formation of some defects in the *n*–In_2_O_3_ layer. As can be seen from the comparison of curves 2 and 4 with curves 1 and 3, 48 h after the first measurement cycle, the factors that influenced the decrease in the photocurrent as the number of bends increased are maintained.

Since the dependencies of the photocurrent on the number of bending cycles for heterojunctions and photoresistors are similar, we can state that the factors leading to the decrease in the photocurrent in these devices are characteristic for InSe:Cd plates. Obviously, upon bending, internal stresses are formed in the InSe layer, which can lead to a change in the forbidden bandwidth and, thus, in the photoresponse bandwidth. In works [58,59], the elastic constants and the pressure dependence of the energy band gap in the lamellar compounds GaS, GaSe, and InSe were studied, from which it can be seen that the compressive forces between the packages are slightly decreasing from GaSe to InSe, from 6.28 × 10^−10^ to 4.8 × 10^−10^ N m^−2^ respectively, while the absorption edge slightly shifts toward higher energies as the pressure increases.

Consequently, one can state that bending by ~15° will exert a weak influence on the PR bandwidth of the *p*–InSe:Cd/*n*–In_2_O_3_ heterojunctions. As can be seen from Figure 5, the photosensitivity band of these heterojunctions covers a broad spectral range, from 1.25 to 3.70 eV. The flexible photodetectors studied in works [13,14] show photosensitivity in the UV-C region (200–300) nm.

## 4. Conclusions

By heat treatment in air, at a temperature of 600 °C, of InSe single-crystalline plates doped with Cd and Sn, in a water-vapor- and oxygen-enriched atmosphere, In_2_O_3_ layers and *p* (*n*)–InSe/*n*–In_2_O_3_ structures can be obtained, displaying blue–orange PL and broadband photosensitivity. As Raman and EDX analyses show, the layer of material formed on the surface of InSe:Sn plates is comprised of the compound In_2_O_3_ with an excess of absorbed oxygen.

The band gap of the native oxide layer, determined by wavelength-modulated reflectance spectroscopy, is equal to 3.57 and 3.67 eV for the In_2_O_3_ layer formed by the heat treatment of single-crystalline InSe plates doped with Sn and Cd, respectively.

The In_2_O_3_ layer is a photoluminescent material in the visible range. The structure of its PL spectrum is determined by actual dopants: Cd in *p*–InSe:Cd/*n*–In_2_O_3_ and Sn in *n*–InSe:Sn/*n^+^*–In_2_O_3_ structures.

The *p*–InSe/*n*–In_2_O_3_ heterojunctions and *p*–InSe:Cd photoresistors maintain their photosensitivity upon multiple bending cycles.

The relative photosensitivity band of *p*–InSe/*n*–In_2_O_3_ structures is determined by the fundamental absorption threshold of *p*–InSe and at higher energies, by that of In_2_O_3_.

## Figures and Tables

**Table 1:** Raman frequencies of the In_2_O_3_ layer obtained by heat treatment in air, at 600 °C, for 6 h, of single-crystalline InSe plates doped with Sn (InSe:Sn/In_2_O_3_ structures), compared to those in In_2_O_3_ and InSe.

No.	Experimental Data		In_2_O_3_ Nanowires [28]	In_2_O_3_ Nanocubes [26]	In_2_O_3_ Bulk [23]	SnO_2_ [30]	InSe [27]
	$\tilde{\nu }$ (cm^−1^)	I, Arb. Units	$\tilde{\nu }$ (cm^−1^)	$\tilde{\nu }$ (cm^−1^)	$\tilde{\nu }$ (cm^−1^)	$\tilde{\nu }$ (cm^−1^)	$\tilde{\nu }$ (cm^−1^)
1.	110	956	109	103	109	-	-
2.	135	970	133	130	131	-	-
3.	216	915	-	-	169	-	199
4.	230	940	231	-	212	225	225
5.	250	979	-	-	-	-	-
6.	306	892	304	302	306	-	407/423
7.	470	813	-	-	-	475	
8.	501	828	-	494	495	-	-
9.	540	798	-	-	-	540	
10.	627	804	-	620	629	-	-
11.	638	800	-	-	-	635	-

## Data Availability

Not applicable.

## References

[1] Himani A., Artur E. (2021). Recent progress in contact, mobility, and encapsulation engineering of InSe and GaSe. InfoMat.

[2] Bucher E. (1992). Photovoltaic properties of solid state junctions of layered semiconductors. Photoelectrochemistry and Photovoltaics of Layered Semiconductors.

[3] Elumalai N.K., Vijila C., Jose R., Uddin A., Ramakrishna S. (2015). Metal oxide semiconducting interfacial layers for photovoltaic and photocatalytic applications. Mater. Renew. Sustain. Energy.

[4] Siciliano T., Di Giulio M., Tepore M., Genga A., Micocci G., Tepore A. (2012). In_2_O_3_ films prepared by thermal oxidation of amorphous InSe thin films. Thin Solid Film..

[5] Vatavu E., Leontie L., Caraman I., Sprincean V., Untila D., Doroftei C., Caraman M. (2020). Optical and structural properties of *n*− and *p*−InSe/In_2_O_3_ heterostructures. J. Lumin..

[6] Liviu L., Sprincean V., Untila D., Spalatu N., Caraman I., Cojocaru A., Susu O., Lupan O., Evtodiev I., Vatavu E. (2019). Synthesis and optical properties of Ga_2_O_3_ nanowires grown on GaS substrate. Thin Solid Film..

[7] Walsh A., Da Silva J.L.F., Wei S.-H., Körber C., Klein A., Piper L.F.J., DeMasi A., Smith K.E., Panaccione G., Torelli P. (2008). Nature of the band gap of In_2_O_3_ revealed by first-principles calculations and X-ray spectroscopy. Phys. Rev. Lett..

[8] Cui J., Wang A., Edleman N.L., Ni J., Lee P., Armstrong N.R., Marks T.J. (2001). Indium Tin Oxide Alternatives–High Work Function Transparent Conducting Oxides as Anodes for Organic Light-Emitting Diodes. Adv. Mater..

[9] Kumar A.S., Wang M., Li Y., Fujita R., Gao X.P.A. (2020). Interfacial Charge Transfer and Gate-Induced Hysteresis in Monochalcogenide InSe/GaSe Heterostructures. ACS Appl. Mater. Inter..

[10] Kohjiro H., Kazuhiro S., Hironori A. (2000). Semiconductor-sensitized solar cells based on nanocrystalline In_2_S_3_/In_2_O_3_ thin film electrodes. Sol. Energy Mat. Sol. Cells.

[11] Afaneh T., Fryer A., Xin Y., Hyde R.H., Kapuruge N., Gutiérrez H.R. (2020). Large-area growth and stability of monolayer gallium monochalcogenides for optoelectronic devices. ACS Appl. Mater. Int..

[12] Shunli W., Sun H., Wang Z., Zeng X., Ungar G., Guo D., Shen J., Li P., Liu A., Li C. (2019). In situ synthesis of monoclinic *β*-Ga_2_O_3_ nanowires on flexible substrate and solar-blind photodetector. J. Alloy. Compd..

[13] Sui Y., Liang H., Huo W., Wang Y., Mei Z. (2020). A flexible and transparent *β*-Ga_2_O_3_ solar-blind ultraviolet photodetector on mica. J. Phys. D Appl. Phys..

[14] Wang Y., Yang Z., Li H., Li S., Zhi Y., Yan Z., Huang X., Wei X., Tang W., Wu Z. (2020). Ultrasensitive flexible solar-blind photodetectors based on graphene/amorphous Ga_2_O_3_ van der Waals heterojunctions. ACS Appl. Mater. Int..

[15] Sen-Tsun J., Yung-Chiun H. (2010). Growth mechanism and photoluminescence properties of In_2_O_3_ nanotowers. Cryst. Growth Des..

[16] Xie C., Mak C., Tao X., Yan F. (2017). Photodetectors based on two-dimensional layered materials beyond graphene. Adv. Funct. Mater..

[17] Jiang Z.X., Wu Z.Y., Ma C.C., Deng J.N., Zhang H., Xu Y., Ye J.D., Fang Z.L., Zhang G.Q., Kang J.Y. (2020). *P*-type *β*-Ga_2_O_3_ metal-semiconductor-metal solar-blind photodetectors with extremely high responsivity and gain-bandwidth product. Mater. Today Phys..

[18] López I., Castaldini A., Cavallini A., Nogales E., Méndez B., Piqueras J. (2014). *β*-Ga_2_O_3_ nanowires for an ultraviolet light selective frequency photodetector. J. Phys. D Appl. Phys..

[19] Ghosh P.K. (1983). Introduction to photoelectron spectroscopy. Chemical Analysis: A Series of Monographs on Analytical Chemistry and Its Applications.

[20] Komolov A.S., Lazneva E.F., Gerasimova N.B., Panina Y.A., Sobolev V.S., Koroleva A.V., Pshenichnyuk S.A., Asfandiarov N.L., Modelli A., Handkee B. (2019). Conduction band electronic states of ultrathin layers of thiophene/phenylene co-oligomers on an oxidized silicon surface. J. Electron. Spectros..

[21] Guo Z., Liu1 J., Jia Y., Chen X., Meng F., Li M., Liu J. (2008). Template synthesis, organic gas-sensing and optical properties of hollow and porous In_2_O_3_ nanospheres. Nanotechnology.

[22] Casey P.S., Rossouw C.J., Boskovic S., Lawrence K.A., Turney T.W. (2006). Incorporation of dopants into the lattice of ZnO nanoparticles to control photoactivity. Superlattice. Microst..

[23] Xie C., Lu X.-T., Ma M.-R., Tong X.-W., Zhang Z.-X., Wang L., Wu C.-Y., Yang W.-H., Luo L.-B. (2019). Catalyst-Free Vapor–Solid Deposition Growth of *β*–Ga_2_O_3_ Nanowires for DUV Photodetector and Image Sensor Application. Adv. Opt. Mater..

[24] Pang H.-F. (2017). Biuret-assisted formation of nanostructured In_2_O_3_ architectures and their photoluminescence properties. J. Lumin..

[25] Kanaya K., Okayama S. (1972). Penetration and energy-loss theory of electrons in solid targets. J. Phys. D Appl. Phys..

[26] Zhu H., Wang X., Yang F., Yang X. (2008). Template-free, surfactantless route to fabricate In(OH)_3_ monocrystalline nanoarchitectures and their conversion to In_2_O_3_. Cryst. Growth Des..

[27] Dussan S., Singh M.K., Kumar A., Katiyar R.S. (2011). Synthesis, Structural and Magnetic Properties of Ni-Doped In_2_O_3_ Nanoparticles. Integr. Ferroelectr..

[28] Caracas R., Cohen R.E. (2007). Post-perovskite phase in selected sesquioxides from density-functional calculations. Phys. Rev. B.

[29] Manmeet K., Jain N., Sharma K., Bhattacharya S., Roy M., Tyagi A.K., Gupta S.K., Yakhmi J.V. (2008). Room-temperature H_2_S gas sensing at ppb level by single crystal In_2_O_3_ whiskers. Sens. Actuat. B-Chem..

[30] Jiayong G., Lu X., Wu J., Xie S., Zhai T., Yu M., Zhang Z., Mao Y., Wang S.C.I., Shen Y. (2013). Oxygen vacancies promoting photoelectrochemical performance of In_2_O_3_ nanocubes. Sci. Rep..

[31] Mahmoud Z., Jain K.P., Mavi H.S., Balkanski M., Julien C., Chevy A. (1996). Raman investigation of InSe doped with GaS. Mater. Sci. Eng. B-Adv..

[32] Kim H.S., Na H.G., Yang J.C., Lee C., Kim H.W. (2011). Synthesis, structure, photoluminescence, and raman spectrum of indium oxide nanowires. Acta Phys. Pol. A.

[33] Thlel B., Helbig R. (1976). Growth of SnO_2_ single crystals by a vapour phase reaction method. J. Cryst. Growth.

[34] Yu P.Y., Cardona M. (2010). Fundamentals of Semiconductors: Physics and Materials Properties.

[35] Savitzky A., Golay M.J.E. (1964). Smoothing and differentiation of data by simplified least squares procedures. Anal. Chem..

[36] Zelewski S.J., Kudrawiec R. (2017). Photoacoustic and modulated reflectance studies of indirect and direct band gap in van der Waals crystals. Sci. Rep..

[37] Mergel D., Qiao Z. (2002). Dielectric modelling of optical spectra of thin In_2_O_3_: Sn films. J. Phys. D Appl. Phys..

[38] Pollak F.H., Balkanski M. (1994). Modulation spectroscopy of semiconductors and semiconductor microstructures. Handbook on Semiconductors, Optical Properties of Semiconductors.

[39] Sell D.D., Stokowski S.E. Modulated piezo reflectance and reflectance studies of GaAs. Modulated piezo reflectance and reflectance studies of GaAs. Proceedings of the Tenth International Conference on the Physics of Semiconductors.

[40] Lang O., Pettenkofer C. (1999). Thin film growth and band lineup of In_2_O_3_ on the layered semiconductor InSe. Int. J. Appl. Phys..

[41] Feneberg M., Nixdorf J., Lidig C., Goldhahn R. (2016). Many-electron effects on the dielectric function of cubic In_2_O_3_: Effective electron mass, band nonparabolicity, band gap renormalization, and Burstein-Moss shift. Phys. Rev. B Condens. Matter.

[42] Karlheinz S. (2013). Semiconductor Physics: An Introduction.

[43] Wasserman A.L. (2005). Band-Structure Effective Mass. Encyclopedia of Condensed Matter Physics.

[44] Fuchs F., Bechstedt F. (2008). Indium-oxide polymorphs from first principles: Quasiparticle electronic states. Phys. Rev. B.

[45] Jeong J.S., Lee J.Y., Lee C.J., An S.J., Yi G.-C. (2004). Synthesis and characterization of high-quality In_2_O_3_ nanobelts via catalyst-free growth using a simple physical vapor deposition at low temperature. Chem. Phys. Lett..

[46] Tao G., Taihong W. (2006). Catalytic growth of In_2_O_3_ nanobelts by vapor transport. J. Cryst. Growth.

[47] Jun T., Naruo C., Yamamoto Y., Matsuoka M. (2002). Sensing properties to dilute chlorine gas of indium oxide based thin film sensors prepared by electron beam evaporation. Sens. Actuat. B-Chem..

[48] Qingsheng L., Lu W., Ma A., Tang J., Lin J., Fang J. (2005). Study of quasi-monodisperse In_2_O_3_ nanocrystals: Synthesis and optical determination. J. Am. Chem. Soc..

[49] Fanhao Z., Zhang X., Wang J., Wang L., Zhang L. (2004). Large-scale growth of In_2_O_3_ nanowires and their optical properties. Nanotechnology.

[50] Camassel J., Merle P., Mathieu H., Chevy A. (1978). Excitonic absorption edge of indium selenide. Phys. Rev. B.

[51] Shigetomi S., Ikari T. (2003). Electrical and optical properties of *n*-and *p*-InSe doped with Sn and As. Int. J. Appl. Phys..

[52] Makkawi O., Huang Y., Feng W., Liu G., Qiu Y., Hu P.A. (2016). Modulation of opto-electronic properties of InSe thin layers via phase transformation. RSC Adv..

[53] Kudrynskyi Z., Khomyak V., Katerynchuk V., Kovalyuk M., Netyaga V., Kushnir B. (2015). Fabrication and characterization of photosensitive *n*-ZnO/*p*-InSe heterojunctions. Thin Solid Film..

[54] Savchyn V.P., Kytsai V.B. (2000). Photoelectric properties of heterostructures based on thermo-oxidated GaSe and InSe crystals. Thin Solid Film..

[55] Katerynchuk V.N., Kovalyuk Z.D. (2004). Fabrication of oxide/*p*–InSe heterostructures with improved photoelectric characteristics. Fiz. Tekh. Poluprovodn..

[56] Il’chuk G.A., Kusznezh V.V., Petrus’ R.Y., Rud’ V.Y., Rud’ Y.V., Terukov E.I., Ukrainets V.O. (2006). Heterophotocells *n*–Ox/*n*–InSe: Creation and properties. Fiz. Tekh. Poluprovodn..

[57] Kovalyuk Z.D., Sydor O.N., Katerynchuk V.N., Netyaga V.V. (2007). Studies of isotype photosensitive heterostructures (intrinsic oxide)/*n*–InSe obtained by long-term thermal oxidation. Fiz. Tekh. Poluprovodn..

[58] Gatulle M., Fischer M. (1984). Elastic Constants of the Layered Compounds GaS, GaSe, InSe, and Their Pressure Dependence II. Theoretical Part. Phys. Status Solidi B.

[59] Gauthier M., Polian A., Besson J.M., Chevy A. (1990). Pressure effect on a layer compound: GaSe. High Press. Res..

